# A positive Selection *Escherichia Coli* Recombinant Protein Expression Vector for One-Step Cloning

**DOI:** 10.3389/fbioe.2021.776828

**Published:** 2022-01-03

**Authors:** Shinto James, Vikas Jain

**Affiliations:** Microbiology and Molecular Biology Laboratory, Department of Biological Sciences, Indian Institute of Science Education and Research (IISER), Bhopal, India

**Keywords:** recombinant protein expression, *E. coli* vector, blunt end cloning, green fluorescent protein (GFP), one-step cloning

## Abstract

We introduce OLIVAR (***O*** rientation se ***L*** ection of ***I*** nsert in ***V*** ector through ***A*** ntisense ***R*** eporter) as a novel selection strategy for the insertion of protein-coding genes into vector backbones. As a proof-of-concept, we have engineered a plasmid vector, pGRASS (***G*** reen fluorescent protein ***R*** eporter from ***A*** ntisense promoter-based ***S*** creening ***S*** ystem), for gene cloning in *E. coli*. With pGRASS, positive clones can be effortlessly distinguished from negative clones after blunt-end cloning. The vector not only screens clones with an insert but also for its correct orientation. The design further allows for the expression of recombinant protein from the T7 promoter in an appropriate host bacterium. With this vector, we are able to reduce the entire cloning workflow into a single step involving a 2-h reaction at room temperature. We believe that our cloning-cum-screening system presented here is extremely cost-effective and straightforward and can be applied to other vector systems and domains such as phage display and library construction.

## Introduction

From the study of gene function to the industrial production of therapeutic proteins, molecular cloning followed by recombinant protein expression is pivotal to many molecular biology investigations and translational applications. The protein-coding gene is first inserted into a vector backbone. The assembled construct is then introduced to an appropriate host organism, where the recombinant DNA replicates concurrently with the host cells. Protein expression is induced in these cells after achieving desired growth (Sambrook and Russell 2006).

Insertion of the gene of interest into a vector backbone can be achieved through various means, and commercial kits are available for rapid cloning applications (Holton and Graham 1991; Hartley et al., 2000; Engler et al., 2008; Gibson et al., 2009; Irwin et al., 2012). However, conventional restriction digestion-based cloning, owing to its cost-effectiveness and relative simplicity, remains the most employed method of cloning. In particular, blunt-end-digestion-based cloning is extremely straightforward and versatile and requires the least amount of planning (Singh and Jain, 2013). Nonetheless, the low efficiency of this technique necessitates further modifications such as dephosphorylation of vector, phosphorylation of inserts as well as to carefully adjust insert to vector ratios to enrich for recombinants. Additionally, extensive screening for desired recombinants is also required after cloning, thus making the entire process time-consuming.

We reasoned that engineering a selection cassette into a vector would eliminate the problem with the low-efficiency of restriction digestion-based cloning since positive clones can be easily picked out from negative clones, even if they are vastly outnumbered. However, most positive selection vectors available only help in cloning the target DNA (Choi et al., 2002). The overexpression of the cloned gene with these vectors is not possible because while they contain sequences for their replication and selection, they lack the necessary elements for target gene expression. A few selection vectors which also allow expression of the cloned gene are also available (Haag and Ostermeier 2009; Banerjee et al., 2010; Prosser et al., 2015; Haustant et al., 2016; Wu et al., 2018) but have not found wide-scale application in routine cloning due to the limitations in their designs. Some of these methods (Haag and Ostermeier 2009; Prosser et al., 2015) require the insert to be supplemented with long additional sequences through PCR to enable selection, whereas others require specific *E. coli* strains (Banerjee et al., 2010), or only allow periplasmic expression of the cloned gene (Haustant et al., 2016). Moreover, in several of these cases (Banerjee et al., 2010; Prosser et al., 2015; Wu et al., 2018), both the reporter and the recombinant protein are expressed from the same promoter. This limits the strength of the promoter that can be employed in these systems since expression from a strong promoter will significantly slow down the growth of host cells on LB-agar plates, making selection difficult. Furthermore, most of these systems require modified primers, digestion of insert and vector with multiple restriction enzymes, and subsequent gel purification of backbone for avoiding false positives, thus having no advantage over traditional methods in terms of time and cost required for cloning.

Here we introduce a novel selection strategy, OLIVAR (for ***O*** rientation se ***L*** ection of ***I*** nsert in ***V*** ector through ***A*** ntisense ***R*** eporter), which overcomes all the drawbacks of the aforementioned cloning and screening methods. We demonstrate that the positive selection expression vector pGRASS (for ***G*** FP ***R*** eporter from ***A*** ntisense promoter-based ***S*** creening ***S*** ystem), developed based on this strategy, allows for the direct screening of positive clones on LB-agar plate. Furthermore, using the one-step cloning methodology proposed here, cloning can be carried out in *E. coli* without any post-PCR modifications of the insert followed by highly efficient screening and high-level protein expression.

## Materials and Methods

### Plasmids, Bacterial Strains, Media, and Growth Conditions

pET3b expression vector was obtained from Novagen. Cloning and screening were carried out in *E. coli* strain XL1-Blue (Stratagene), whereas protein expression was done in *E. coli* strain BL21 (DE3) (Lucigen). Both strains were grown in LB broth (Difco) supplemented with 100 μg/ml Ampicillin, and at 37 °C with constant shaking at 200 rpm. Bacterial growth on solid medium was carried out on LB broth supplemented with 1.5% agar and ampicillin.

### Reagents and Deoxyribonucleic Acid Synthesis

Restriction enzymes, T4 DNA ligase, Antarctic phosphatase, T4 polynucleotide kinase, and Phusion HF polymerase were procured from NEB and were used following the manufacturer’s instructions. Phusion polymerase was used in all PCR reactions using oligos listed in Supplementary Table S2. Fast DNA End Repair Kit was purchased from Thermo Fisher Scientific. DNA oligos were obtained from Macrogen (South Korea). The 1 kb selection-expression cassette was synthesized by Genewiz (United States). All the reagents were purchased from Sigma Aldrich.

### Cloning in pGRASS

Cloning in pGASS was carried out as one-step or two-step depending on if the gene of interest contained a SmaI site. For two-step cloning, digestion of plasmid was followed by cleanup to remove the enzyme. This was performed when the gene to be inserted also contained a SmaI site. 4 μg of plasmid was digested with SmaI following the manufacturer’s instructions. The digestion reaction was either cleaned up (Qiagen) or the enzyme was heat-inactivated according to manufacturer’s instructions. The digested vector was used for all ligation reactions. For one-step cloning, plasmid and insert DNA together were incubated with four units of SmaI and six units of T4 DNA ligase at 22 °C for 2 h. 5–10 ng of plasmid backbone was incubated with 50–150 ng of insert for the ligation reactions. All the ligation reactions were used for *E. coli* XL1-Blue transformation. For screening, colony PCR (cPCR) was carried out essentially as previously described (Singh and Jain, 2013; Dubey et al., 2016) using the oligos listed in Supplementary Table S2.

### Quantitative Polymerase Chain Reaction

Copy number of pGRASS(*lacO-*) *ori* mutant was determined using comparative C_t_ method (ΔΔC_t_) with quantitative PCR (qPCR) using the oligos listed in Supplementary Table S2. pET3b plasmid was used as the reference sample. Primers were designed to target a common sequence in both the plasmids. The plasmids were introduced in *E. coli* BL21 (DE3). A single copy gene (*umuD*) in host genome was used as the endogenous target. 1 mL of secondary culture was harvested at OD600 ∼0.6, followed by resuspension of cells in MilliQ water. The resuspended cells were lysed by boiling at 100 °C for 10 min, followed by centrifugation for 15 min at 14,000 × g at RT. The supernatant was then diluted 100 fold and was used as template for qPCR. Relative copy number was calculated by the equation $RQ=E_{p}^{\Delta C_{t\_pls}}E_{c}^{\Delta C_{t\_chr}}$ , where E_p_ is the efficiency of the amplification of the plasmid target, E_c_ is the efficiency of amplification from the chromosome target, ΔC_t_pls_ is the difference in C_t_ values between the reference and test samples for the plasmid target, and ΔC_t_chr_ is the difference in C_t_ values between reference and test samples for the chromosome target. E_p_ and E_c_ were calculated by sigmoidal fitting of the respective qPCR data in a four-parameter sigmoidal model using the qPCR package in the R computing environment available at http://www.dr-spiess.de/qpcR.html

### Protein Expression Analysis

Recombinant protein expression from the T7 promoter was assessed by following the method as described elsewhere (Dubey et al., 2016). Cells carrying either pET21b or pMS_QS_CHS vector (Singh and Jain, 2013) with the same gene were taken as positive controls.

### *In Silico* Analysis

All DNA constructs were designed using the SnapGene software (Insightful Science; snapgene.com). The same platform was used to simulate cloning and other modifications in DNA. Stem-loops in 5′ leader sequence transcripts were analyzed using CentroidFold available at http://rtools.cbrc.jp/centroidfold. To determine the fraction of genes containing the codons TCA, TTA, and CTA, the coding sequence (CDS) datasets of the desired organisms were downloaded from NCBI assembly database available at https://www.ncbi.nlm.nih.gov/assembly. Each dataset was separately analyzed for the fraction of genes containing target codons in their open reading frames with a code written in python programming language. The source code is available at https://github.com/shintojames1/gene-specific-codon-frequency.

## Results

### The OLIVAR Strategy and the Designing of pGRASS Vector

**FIGURE 1:**
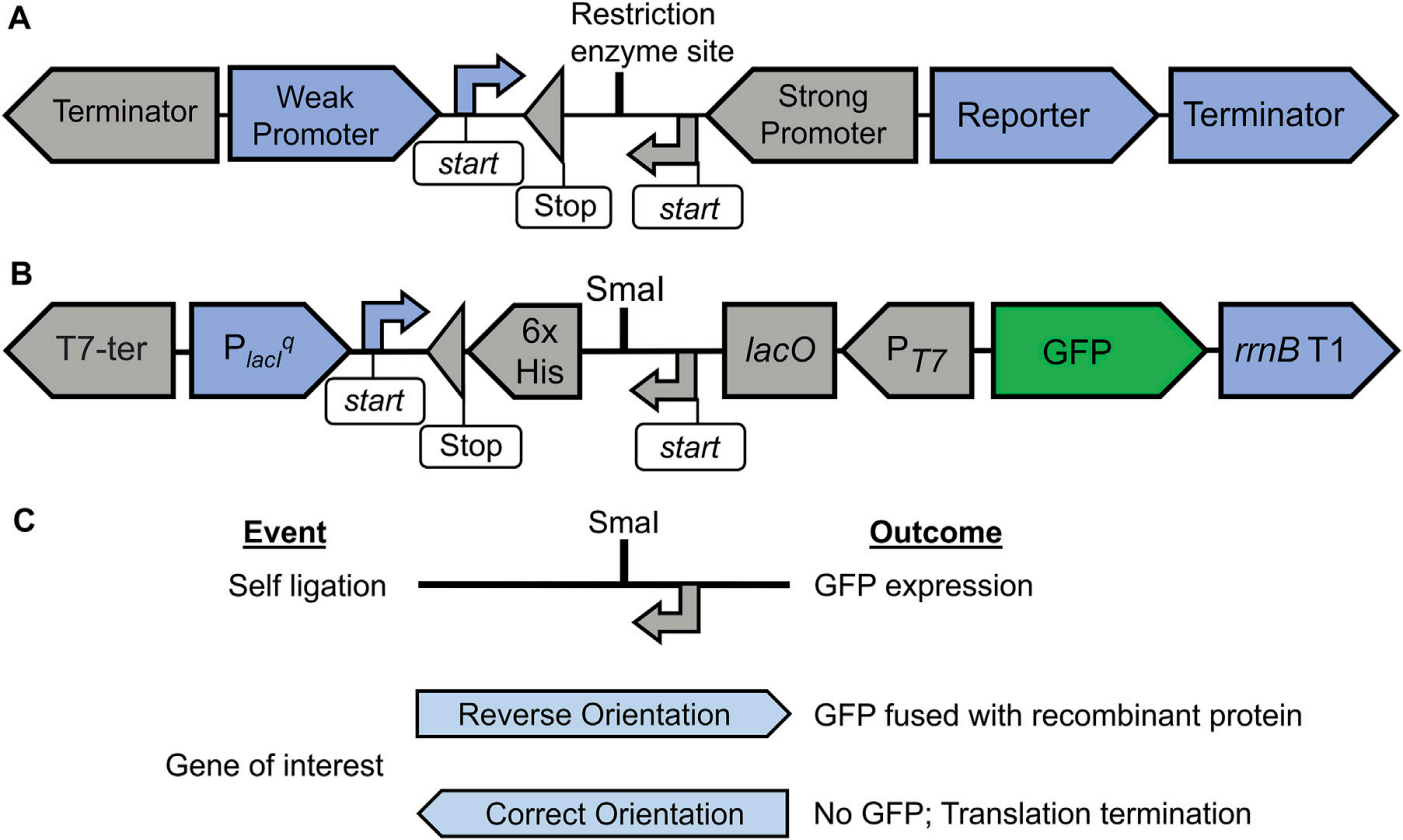
Schematic representation of OLIVAR-based screening strategy and the construction of pGRASS vector. Panel **(A)** depicts the OLIVAR system. The design consists of a selection cassette (blue) and an expression cassette (grey) placed in opposing transcription orientations. The selection cassette consists of a weak promoter, a reporter, and a transcription terminator. The blue arrow “start” represents the translation start site that initiates translation for the reporter. The expression cassette consists of a strong promoter along with a transcription terminator. The grey coloured “start” and “stop” signals are for the translation. Restriction enzyme site allows for the insertion of the DNA. Panel **(B)** shows the Selection-cum-expression cassette of the pGRASS vector. The screening cassette contains a weak lacI^q^ promoter (P_lacI_^q^), GFP reporter, and the rrnB T1 transcription terminator. The expression cassette contains T7 promoter (P_T7_), lac operator (lacO), codons for hexa-histidine tag (6x His), and the T7 transcription terminator. A SmaI site is present that allows for the cloning of a target gene. The start and stop signals are exactly as panel **(A)**. Panel **(C)** depicts the screening strategy in pGRASS vector. Ligation reaction can lead to one of the three events and outcomes as shown. Self-ligation event and the insertion of gene of interest in reverse orientation will result in GFP production, whereas ligation of gene of interest in correct orientation will not result in GFP production. The orientation of gene of interest (reverse or correct) is with respect to P_T7_.

We present here our newly developed methodology, OLIVAR, wherein the two cassettes, *viz*. the selection cassette (SC) and the target gene expression cassette (EC), are integrated together but placed in the opposite orientation (Figure 1A). The SC is formed with a weak promoter that drives the expression of a reporter gene, whereas the EC contains a strong promoter for the target gene expression and is integrated inside the SC in the antisense-transcribing direction. The methodology allows for the screening of positive or recombinant clones in which the gene of interest is present in the EC in the correct orientation.

Based on this methodology, we have engineered pGRASS vector that allows for the positive screening of the recombinant plasmid (Figure 1B, Supplementary Figure S1). In this vector, the SC contains a *lacI* ^q^ promoter (P_*lacI*_^*q*^) that drives the expression of the reporter GFP. The EC contains a T7 promoter (P_T7_), which drives the target gene expression. The EC also contains SmaI restriction enzyme site, which is located downstream of the translation start site for the target protein and is used for the cloning of the target gene following blunt-end ligation. Furthermore, the EC also carries codons for hexa-histidine tag that add six histidines in tandem at the C-terminus of the expressed protein. EC sequence has been designed to be devoid of any stop codons in the antisense reading frame so that the translation initiating on the P_*lacI*_^*q*^ transcript can read through this region and express GFP. Thus, in the case of self-ligated clones, where the original vector is reconstituted, GFP is expressed from P_*lacI*_^*q*^, allowing the identification of negative clones (Figure 1C).

Digestion of DNA with SmaI gives rise to blunt ends. Hence, the target gene that is cloned at SmaI site can be inserted in either direction from the P_T7_. The pGRASS vector additionally offers the orientation selection (Figure 1C), provided the target gene is PCR-amplified without a stop codon. Since both SC and EC are in opposite directions, a gene that is inserted in the EC in reverse (undesired) orientation is translated in the correct orientation when transcribed from P_*lacI*_^*q*^, thus leading to GFP expression; this is possible because, in pGRASS vector, both EC and SC have been tailored such that the target gene ORF cloned in the incorrect orientation at SmaI site remain in-frame with GFP ORF. In other words, since the inserted gene is devoid of stop codons, the translation initiating on the P_*lacI*_^*q*^ transcript reads through the gene of interest and then the GFP, producing a fusion protein that contains the cloned protein at the N-terminus of GFP (Figure 1C). Therefore, the clones where the gene of interest is inserted in the reverse orientation also express GFP, which allows for the screening of negative clones carrying insert located in the reverse direction. In all of these cases, GFP expression is monitored by observing and imaging the obtained transformants using blue light (∼400 nm) and GFP filter.

Finally, if the gene of interest inserts in the desired orientation inside the EC, it will be placed in the antisense reading frame in SC. Any of the three codons, namely, TCA, TTA, or CTA, if present in the open reading frame of the target gene, will code for stop codon in the reverse complement reading frame. This will result in the translation termination of the P_*lacI*_^*q*^ transcript before reading GFP codons, leading to no GFP production (Figure 1C) and thus forming non-fluorescing colonies on agar plate. These colonies can be distinguished from the fluorescing colonies in blue light. We additionally discovered that TCA, TTA, and CTA codons are abundantly present across genomes (Supplementary Table S1). Hence, most genes in most organisms can be expected to have one or more of these codons that will facilitate screening in pGRASS.

The transcription and translation initiation signals of EC in pGRASS were taken from the T7 phage gene-10 leader sequence, similar to the pET vectors. However, unlike pET vectors, we excluded additional sequences in the 5′ leader of gene-10 and the *lac* operator to keep the expression cassette minimal. The entire DNA segment containing the EC and SC was commercially synthesized and was inserted into the low copy (LC) number pET3b vector backbone by replacing its expression cassette to generate pGRASS(*lacO*-)LC, which was used next to assess GFP expression in *E. coli*.

### Serendipitous Discovery of High Copy Number pGRASS(*lacO*-) Vector

*E. coli* cells transformed with pGRASS(*lacO*-)LC were all found to express GFP as expected; here, GFP expression is driven by P_*lacI*_^q^. However, we noticed very less GFP fluorescence in the obtained colonies, which was suboptimal for easy distinction on a blue-light transilluminator (offering ∼400 nm). Surprisingly, one of these colonies gave fluorescence higher than the other colonies on the plate. We hypothesized that the higher fluorescence could be because of the occurrence of certain mutations in either GFP or its promoter. Our DNA sequencing data, however, suggested that neither was the case. In order to verify if the increase in fluorescence was because of increase in plasmid copy number, we carried out a quantitative PCR to measure the plasmid copy number. Our data show that indeed, the pGRASS vector has ∼3-fold higher copy number as compared to the wild-type pET3b vector (Supplementary Figure S2). Subsequent sequencing analysis of the origin of replication region revealed a single cytosine nucleotide deletion in the *ori*. Further, this mutation was mapped inside the stem-loop region 2 (SL-2) of RNA I and RNA II transcripts that are involved in the negative feedback regulation of the plasmid copy number (Supplementary Figure S3). Previous studies have noted role of similar mutations in this sequence in altering plasmid copy number in *E. coli* (Lacatena and Cesareni, 1981; Tomizawa and Itoh, 1981). In particular, mutations in the same stem-loop region have been shown to result in a 1.5 to 3.5-fold increase in copy number (Lacatena and Cesareni, 1981). This increase in copy number was suggested to be due to decrease in the RNAI-RNAII interaction energetics resulting from C to T substitution (Lacatena and Cesareni, 1981). Here, we speculate that the mutation in the SL2 region in pGRASS(*lacO*-)LC negatively affects the RNAI-RNAII interaction, which lowers the negative feedback regulation by RNAI, thereby resulting in an increased copy number. Due to the ease of screening from the enhanced GFP fluorescence without increasing the promoter strength, we used this mutated vector for all further experiments. This *ori* mutant is hereafter referred to as pGRASS(*lacO-*).

### Optimization of Target Gene Expression From the T7 Cassette

We next characterized target gene expression from P_T7_. Genes such as *dnaN, adhE2,* and *mdoR* were PCR-amplified from the genomic DNA of *M. smegmatis* and were cloned in the pGRASS(*lacO*-) vector. Positive clones were introduced in *E. coli* BL21 (DE3) and the protein induction profile was examined after IPTG induction. The induction profile showed that the protein expression levels from pGRASS(*lacO*-) were significantly lower compared to the positive controls (Supplementary Figure S4). As a part of troubleshooting, we generated several modifications in our plasmid, where parts were either removed or exchanged with other vectors that offer high expression; protein expression analysis from these constructs revealed that the compromised expression might have resulted from the expression cassette lacking an optimal 5′ leader sequence (data not shown).

**FIGURE 2:**
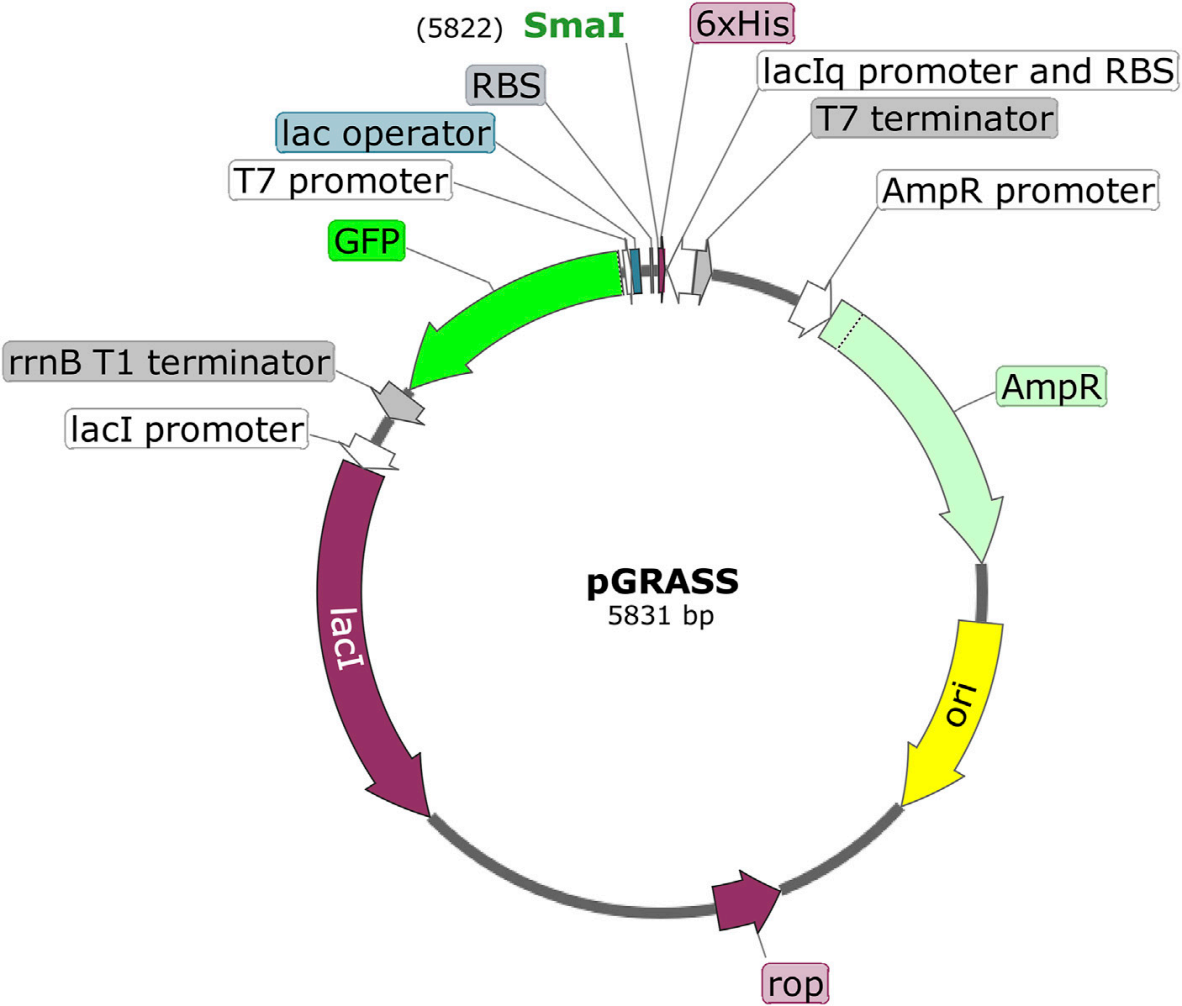
Schematic of pGRASS expression vector. The T7 expression cassette contains T7 promoter, lac operator, ribosome binding site (RBS), SmaI site, codons for hexa-histidine tag (6xHis), and the T7 terminator. The selection cassette contains *lacI* ^q^ promoter (with RBS), GFP reporter, and *rrnB* T1 terminator. The pGRASS vector also contains *lac* repressor-coding gene (*lacI*) under the *lacI* promoter. ‘AmpR’ is the β-lactamase-coding gene. ‘ori’ represents origin of replication in *E. coli*. ‘rop’ codes for ROP protein, which is involved in the regulation of plasmid replication. The orientation of various genes is shown. The total size of the plasmid is given (5,831 bp).

We noticed that most of the vectors of pET series were constructed by inserting -23 to +96 bases from the T7 gene 10 into pBR322 backbone (Studier et al., 1990). Furthermore, 5′ end of this inserted sequence contains a potential stem-loop in the first 21 nucleotides of the T7 transcript, which is suggested to be involved in improving the stability or translatability of the mRNA. Later studies conclusively proved the role of such stem-loops in enhancing the expression of downstream genes in *E. coli* (Arnold et al., 1998; Bricker and Belasco, 1999). However, most vectors developed from pET11 onwards contain a *lac* operator (*lacO*) installed after P_T7_ (Dubendorff and Studier, 1991). This should, in principle, disrupt the stem-loop at the 5′ end of the transcript originating from P_T7_ in these vectors. However, our *in silico* analysis to study the impact of *lacO* in the stem-loop formation showed a stronger stem-loop structure following *lacO* insertion (Supplementary Figure S5), which is probably due to the two-fold symmetry of the *lac* operator sequence (Gilbert and Maxam 1973). Since an optimal leader sequence was found to be important in expressing the downstream gene, we incorporated the entire T7 gene 10 leader sequence along with *lacO* in pGRASS(*lacO*-); Supplementary Figure S6 depicts the entire flowchart of this process. In addition, a *lacI* cassette coding for the Lac repressor was also incorporated into the plasmid so that introduction of plasmid into *E. coli* cells does not titrate out Lac repressor expressing from the genome. This modified plasmid is represented as pGRASS (Figure 2).

### Sample Cloning, Screening, and Protein Expression

**FIGURE 3:**
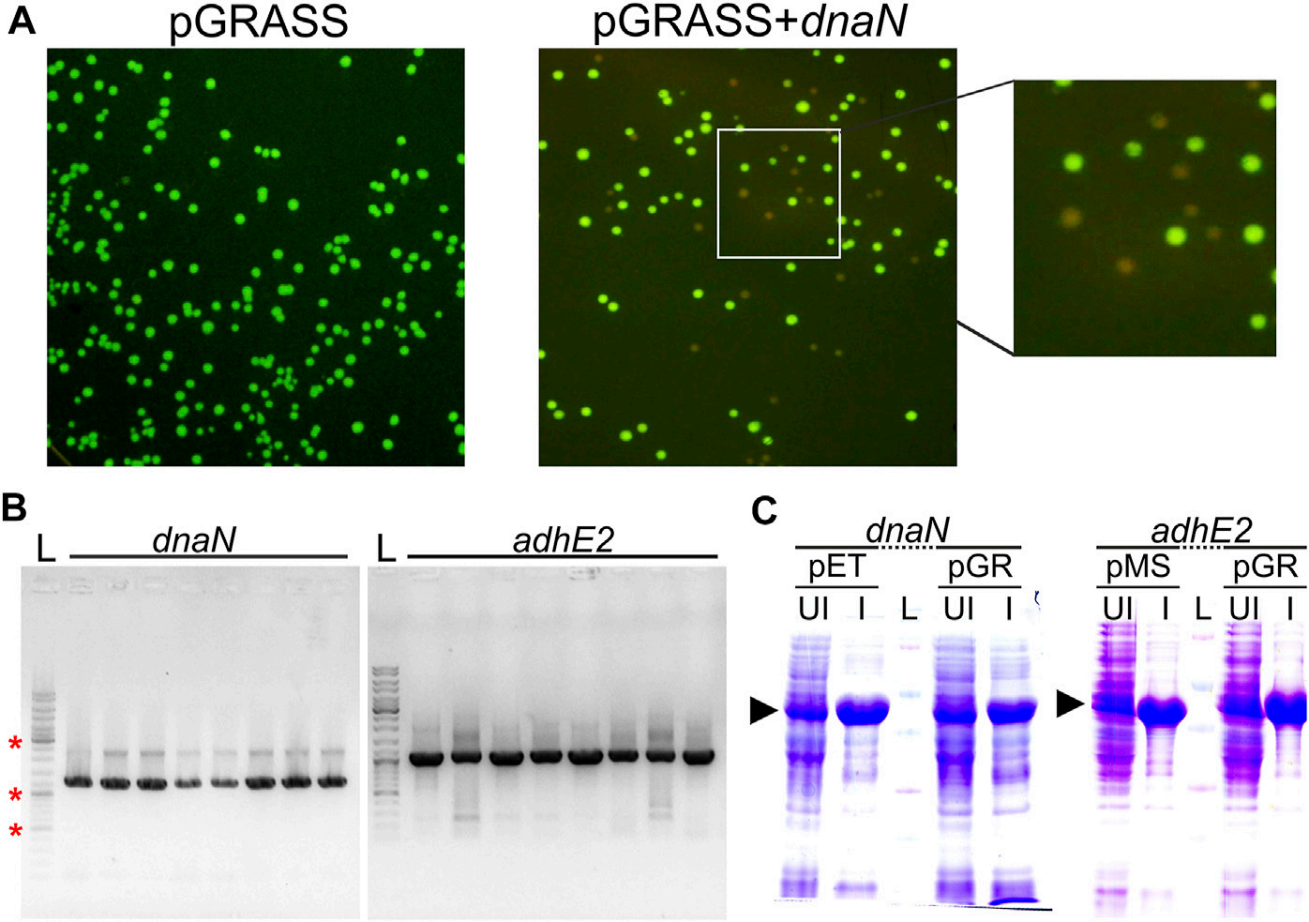
Cloning, screening, and expression of protein coding genes in pGRASS. **(A)** LB-Agar plates having the *E. coli* cells transformed with pGRASS self-ligation control (pGRASS) or ligation sample containing pGRASS vector with *dnaN* insert (pGRASS+*dnaN*), imaged under blue light are shown. The inset on the far right shows the zoomed in section of the pGRASS+*dnaN* plate. **(B)** Colony PCR from eight randomly picked non-fluorescing colonies from pGRASS+*dnaN* and pGRASS+*adhE2* transformation plate. Vector-specific primer was paired with insert-specific primer to assess insert orientation. Labelled as ‘L’ is the DNA ladder with three bands marked with ‘*’ corresponding to 0.5, 1.0, and 3.0 kb. **(C)** Protein expression profiles of DnaN (42 kDa) and AdhE2 (39 kDa). The genes were cloned in pGRASS (pGR) and the expression of these genes is compared with the positive controls; the positive control here corresponds to the gene present in either pET21b (*dnaN*) or pMS vector (*adhE2*) with C-terminal histidine tag in each case. ‘UI’ and ‘I’ represent IPTG uninduced and induced samples, respectively. The protein ladder lane is labelled as ‘L’. The induced protein bands are marked with arrowhead.

We next tested pGRASS vector for cloning of a target gene, screening of positive clones, and protein production. We cloned *dnaN* and *adhE2* from *M. smegmatis* in pGRASS. While the self-ligation control experiment yielded green-fluorescing colonies on the LB agar plate, the ligation samples contained both non-fluorescing and green-fluorescing colonies (Figure 3A). Expectedly, colony PCR (cPCR)-based analysis of the non-fluorescing colonies showed the presence of target gene in the correct orientation (Figure 3B). Next, protein production after IPTG induction was examined to assess if the modifications in the vector helped in improving protein expression. We found that the expression level of the target proteins improved significantly and matched the positive controls (Figure 3C).

**FIGURE 4:**
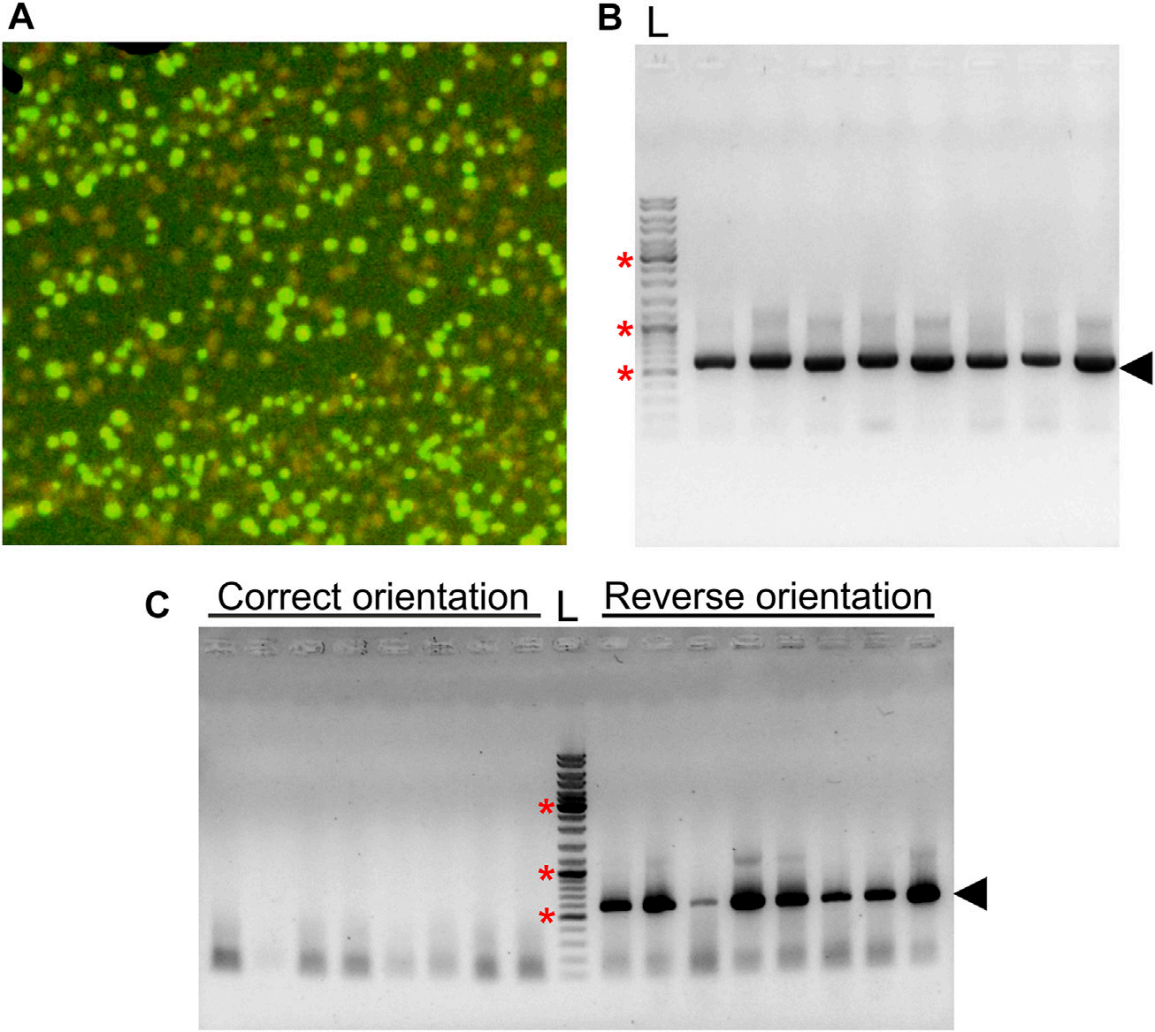
One step cloning of *mdoR* in pGRASS. **(A)** Colonies formed by *E. coli* cells transformed with a ligation sample containing pGRASS vector with *mdoR* insert, imaged under blue light are shown. **(B)** An agarose gel image with the colony PCR result of the non-fluorescing colonies from the plate is shown. Gene forward and vector reverse primers were used for the PCR reaction. Lane ‘L’ represents DNA ladder with few bands marked with ‘*’, corresponding to 0.5, 1.0, and 3.0 kb size. **(C)** Colony PCR of eight randomly selected green fluorescent colonies from the transformation plate. The amplified band is marked with an arrowhead in both panels **(B,C)**.

We also tested our vector for a one-step cloning strategy. SmaI restriction enzyme has been shown to be active in T4 ligase buffer, and the addition of SmaI in the ligation reaction has been carried out previously to enrich recombinants (Liu and Schwartz, 1992). In our experiments, ligation of insert and the vector leads to disruption of SmaI site, whereas a self-ligation event restores it. We reasoned that this feature of blunt-end cloning could be used for a one-step cloning strategy with pGRASS, where digestion and ligation reactions can be carried out in a single-tube, single-step format, provided the gene being cloned is devoid of any SmaI site. To check the applicability of the method, we carried out one-step cloning of *mdoR* gene in pGRASS. We observed that nearly half of the colonies obtained after ligation did not show GFP fluorescence (Figure 4A) and harbored *mdoR* insert in the correct orientation (Figure 4B). Additionally, cPCR from the fluorescing colonies showed that all of them had the *mdoR* insert in the reverse orientation (Figure 4C).

Since our method relies on the presence of specific codons, as described previously, in the target gene to enable selection, it poses a limitation to the usage of our designed vector. Therefore, we asked if pGRASS vector could be used for cloning of those genes that lack such codons. We hypothesized that when an ORF lacking these codons is inserted in the correct orientation, GFP will carry a random, unstructured peptide at its N-terminus, which will lead to the degradation of the synthesized protein in *E. coli* thus resulting in low or non-fluorescing colonies. To test this hypothesis, we cloned the DNA segment corresponding to the N-terminal domain of D29 mycobacteriophage LysA (Pohane et al., 2014), which is devoid of such codons, in pGRASS. As expected, we found several low-fluorescence colonies in our cloning experiment; one such colony after patching is shown in Supplementary Figure S7A. Additionally, colony PCR of such clones revealed that they carried insert in the desired orientation (Supplementary Figure S7B). We, therefore, believe that the pGRASS vector designed here can be used for rapid cloning of most genes for protein production purposes. Furthermore, we are tempted to suggest that pGRASS vector can also be used for the construction of gene expression library of an organism, although the supporting data in this regard are currently lacking.

## Discussion

We describe here a novel genetic design, OLIVAR, which enables the selection of recombinants with the desired orientation of the cloned DNA segment. This strategy was designed to allow direct cloning of PCR-amplified genes without any post-PCR modifications, to keep the cloning procedure straightforward, high-throughput, and near-scarless. OLIVAR is different from the previously described selection systems as it employs premature translation termination of a reporter by virtue of certain codons (TTA, TCA, & CTA) present in the gene to enable selection. Nevertheless, by successful cloning of mycobacteriophage D29 Lysin A N-terminal domain, we show that it is not entirely necessary to have the aforementioned codons in the insert to enable screening, and the formation of an unstructured peptide at the N-terminus of GFP itself is likely sufficient to enable screening. We demonstrate the usefulness of our design by engineering an *E. coli* expression vector, pGRASS, where GFP is used as the reporter and allows for the selection of recombinant clones directly on the LB agar plate.

In pGRASS, the weak constitutive promoter P_*lacI*_^*q*^ opposes the strong and inducible P_T7_. The screening is carried out in *E. coli* lacking T7 polymerase; therefore, the selection cassette functions without any interference from P_T7_. However, during recombinant protein expression, T7 transcription directly opposes P_*lacI*_^*q*^ transcription. At this stage, the interacting promoters can suppress each other’s activity through transcriptional interference (Shearwin et al., 2005; Liu and Kobayashi, 2007) or antisense-RNA mediated repression (Brantl and Wagner, 2002; Brophy and Voigt, 2016). Previous studies have, however, shown that interference between a strong and a weak promoter usually results in the strong promoter repressing the weak promoter, without a significant decrease in the expression from the strong promoter (Sneppen et al., 2005). In particular, a recent study showed that when P_T7_ opposed a weak *E. coli* promoter, expression from *E. coli* promoter drastically reduced, whereas expression from P_T7_ was retained (Hoffmann et al., 2016). Here, we show that T7 RNA Polymerase-mediated expression of the target gene in pGRASS is not significantly affected by P_*lacI*_^*q*^ activity.

Through the blunt-end cloning and expression of genes such as *dnaN, mdoR*, and *adhE2* from *M. smegmatis* in pGRASS, we show that our approach enables reliable screening and offers protein yields similar to the commercial expression vectors. Due to the ease of screening, steps such as dephosphorylation of vector backbone (to reduce self-ligation events) and phosphorylation of insert (required for ligation in a dephosphorylated vector) are dispensable, further simplifying blunt-end cloning. Additionally, since the insert is not phosphorylated, the possibility of multiple inserts in the vector backbone is avoided. This further eliminates the requirement of carefully optimizing the insert to vector ratio before ligation.

Moreover, in pGRASS vector, since the insertion of gene disrupts SmaI restriction site making the ligated construct immune to further digestion, it becomes possible to combine digestion and ligation in a single tube single-step reaction, provided the insert does not contain SmaI site. We demonstrate this with *mdoR* cloning, where the entire cloning was carried out in a 2-h reaction at room temperature.

We conclude that the vector designed here offers a seamless screening system and a versatile cloning system to achieve one-step cloning followed by high-level protein production. Without the requirement of modified primers, insert and backbone preparation, and screening using PCR after cloning, we believe that ours will be the most cost-effective and straightforward method of cloning currently available. We envisage that the OLIVAR strategy can be readily adapted to expression systems in other organisms and even to systems such as pJuFo-based phage display technology (Crameri and Suter, 1993). We strongly believe that our vector will find tremendous applications in routine cloning works in molecular biology and structural biology investigations and in other cloning and expression systems.

## Data Availability

The original contributions presented in the study are included in the article/Supplementary Material. Further inquiries can be directed to the corresponding author.
