# Supplementary material for: A positive Selection Escherichia Coli Recombinant Protein Expression Vector for One-Step Cloning
Source: Front Bioeng Biotechnol. 2022 Jan 3;9:776828. doi: 10.3389/fbioe.2021.776828 (PMC8761972; doi:10.3389/fbioe.2021.776828)
Supplement: Supplementary file 1 [file DataSheet2.PDF]

Supplementary figures

Figure S1:

A

T7 Terminator - LacI^{q} promoter and RBS - Hexa-histidine tag - RBS - Lac operator - T7 promoter - GFP - rrnBT1 Terminator

B

```
CAAAAAACCCCTCAAGACCCGTTTAGAGGCCCCAAGGGGTTATGCTAGATCGACACC
ATCGAATGGTGCAAAACCTTTCGCGGTATGGCATGATAGCGCCCGGAAGAGAGTCAA
TTCAGGGTGGTGAATATGTTAATGGTGATGGTGATGGTGCCCGGGCATATCTATATC
TCCTTCTTAAAGTTAAACAAAATTATTTCTAGAGGGGAATTGTTATCCGCTCACAAT
TCCCCTATAGTGAGTCGTATTAATTTCGCGCATATGAGCAAGGGCGAGGAGCTGTTC
ACCGGGGTGGTGCCCATCCTGGTCGAGCTGGACGGCGACGTAAACGGCCACAAGTTC
AGCGTGCGCGGCGAGGGCGAGGGCGATGCCACCAACGGCAAGCTGACCCTGAAGTTC
ATCTGCACCACCGGCAAGCTGCCCGTGCCCTGGCCCACCCTCGTGACCACCCTGACC
TACGGCGTGCAGTGCTTCAGCCGCTACCCCGACCACATGAAGCAGCACGACTTCTTC
AAGTCCGCCATGCCCGAAGGCTACGTCCAGGAGCGCACCATCTCCTTCAAGGACGAC
GGCACCTACAAGACCCGCGCCGAGGTGAAGTTCGAGGGCGACACCCTGGTGAACCGC
ATCGAGCTGAAGGGCATCGACTTCAAGGAGGACGGCAACATCCTGGGGCACAAGCTG
GAGTACAACTTCAACAGCCACAACGTCTATATCACGGCCGACAAGCAGAAGAACGGC
ATCAAGGCGAACTTCAAGATCCGCCACAACGTCGAGGACGGCAGCGTGCAGCTCGCC
GACCACTACCAGCAGAACACCCCCATCGGCGACGGCCCCGTGCTGCTGCCCGACAAC
CACTACCTGAGCACCCAGTCCAAGCTGAGCAAAGACCCCAACGAGAAGCGCGATCAC
ATGGTCCTGCTGGAGTTCGTGACCGCCGCCGGGATCACTCTCGGCATGGACGAGCTG
TACAAGGTTAACTGAAGATCTAGAAAAACGAAAGGCTCAGTCGAAAGACTGGGCCTT
TCGTTTTATCTGTTGTTTGTCGGTGAACGCTCTCCTGAGTAGGACAAAT
```

Figure S1: The nucleotide sequence of the selection-cum-expression cassette of pGRASS. Panel A shows the arrangement of various elements in the plasmid. Panel B displays the complete sequence of the cassette. Various elements present in the cassette are color coded and labelled with the same color. SmaI restriction site sequence is underlined in panel B.

Figure S2:

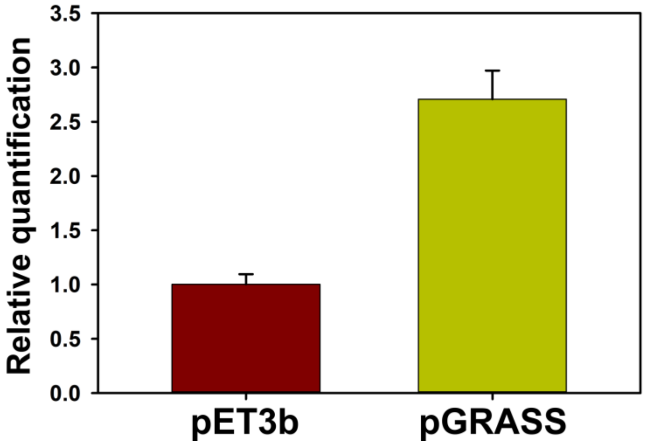

Figure S2: Estimation of the copy number of pGRASS(*lacO*-) *ori* mutant relative to pET3b.

Figure S3:

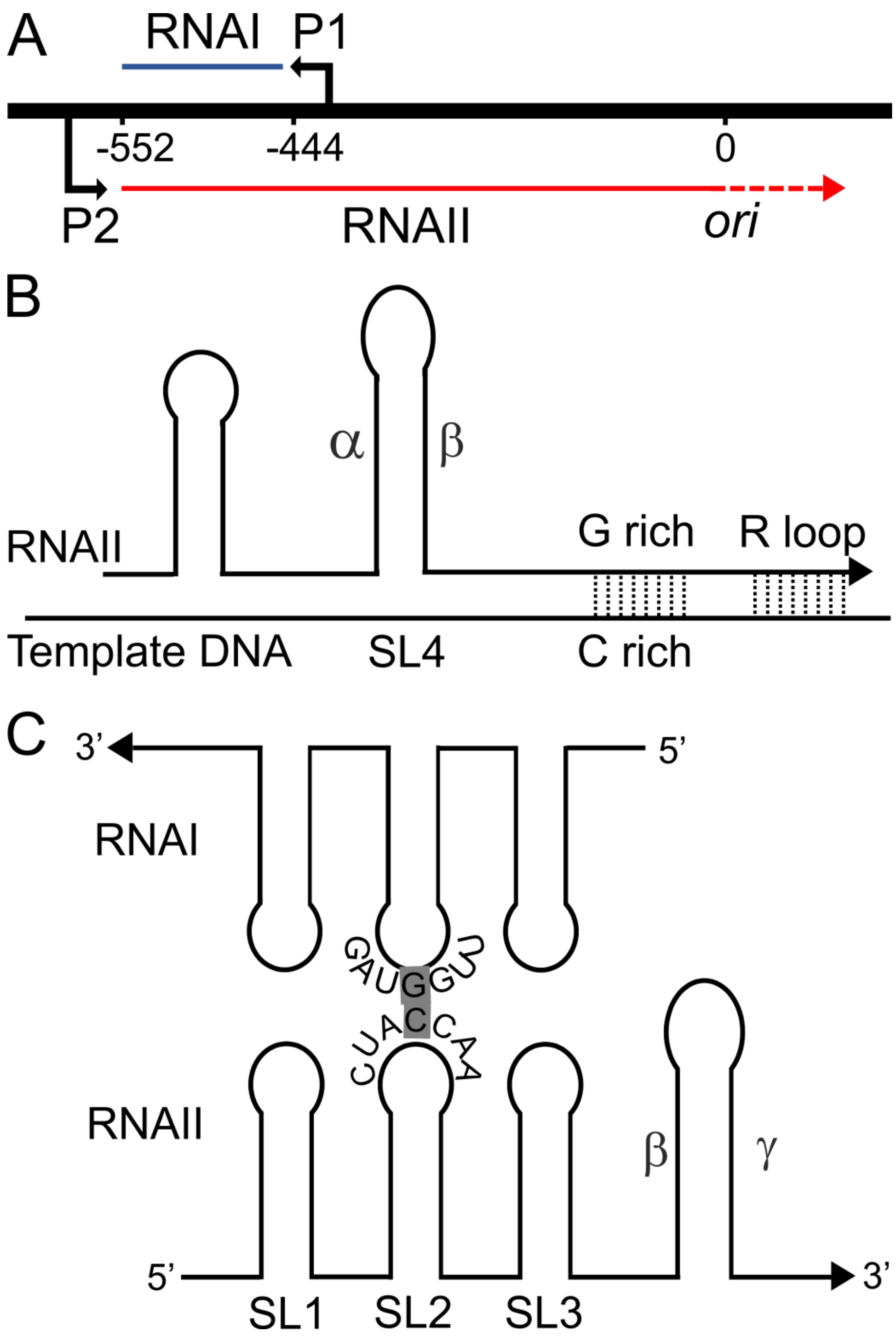

Figure S3: Graphical representation of the pMB1 replication regulation system and the isolated copy number mutant. (A) Schematic of the *ori* region. Here promoter P2 produces RNAII, which acts as a primer for plasmid replication, whereas the antisense promoter P1 expresses RNAI, which acts to suppress plasmid replication. Denoted as 'Ori' is the site where

replication starts and is used as a reference (0) for numbering the plasmid bases. (B) Interaction of the pre-primer RNAII with its DNA template in the *ori* region. The α - β interaction in RNAII results in formation of stem loop 4 (SL4). This allows for the positioning RNAII in the *ori* region enabling interaction of G rich region in RNA II with C rich region in the *ori* resulting in the formation of R-loop. This step is critical for replication initiation. (C) RNAI and RNAII are capable of forming three complimentary stem loops (SL1, SL2, and SL3) as labelled. Both the RNAs then interact with each other through the unpaired nucleotides in the loop region of their three stem loops to form what is called the 'kissing complex'. This interaction breaks the α - β confirmation in RNAII (as shown in panel b) and favours the β - γ confirmation, rendering the RNAII incapable of acting as the pre-primer. Mutations that enhance this interaction act to decrease the copy number and vice versa; the deletion mutation identified in pGRASS is highlighted in the stem-loop 2 (SL2) region.

Figure S4:

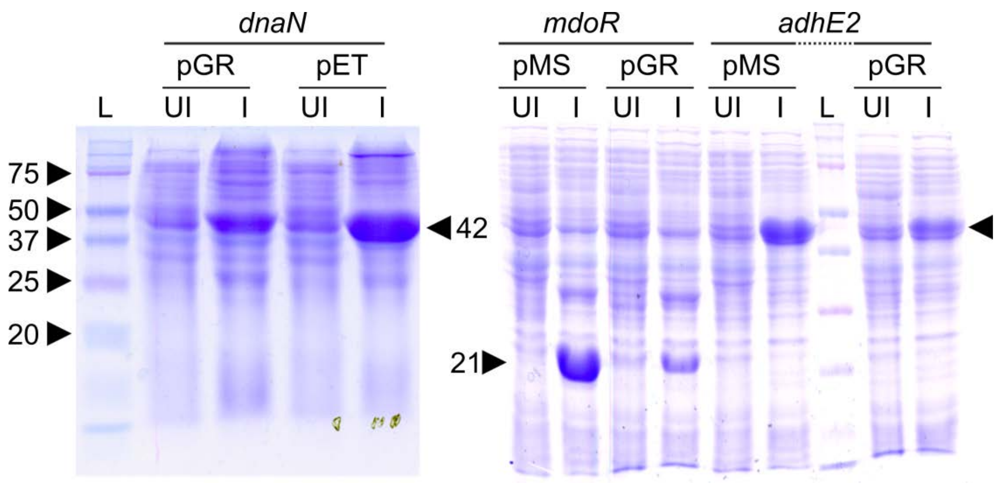

Figure S4: Protein expression analysis DnaN, MdoR, and AdhE2 cloned in pGRASS(*lac*O-). SDS-PAGE profile of the expression of three proteins is shown here. The production of these proteins from their control plasmids is also shown. These include pET21b for DnaN, and pMS-QS-CHS for MdoR and AdhE2. 'UI' and 'I' represent the IPTG uninduced and induced samples, respectively. The protein ladder used is labelled as 'L' with a few bands marked (the values are in kDa). All the overexpressed proteins are also marked with arrowhead along with their sizes in kDa.

Figure S5:

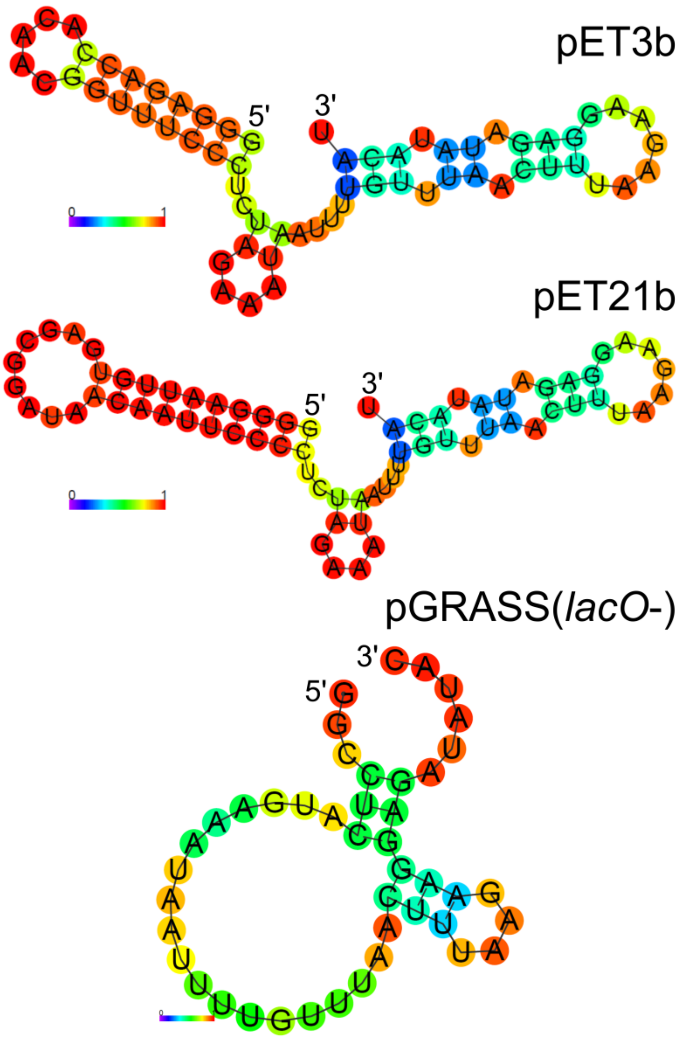

Figure S5: *In silico* analysis of the stem-loop formation in the T7 promoter-initiated transcripts produced from various vectors. The possible stem-loop formation in each case is shown. pET3b transcript contains the T7 gene 10 leader sequence at its 5’ end, whereas pET21b contains an additional *lac* operator insertion at the beginning of the transcript. The T7 transcript from pGRASS(*lac*O-) does not form a stem-loop at its 5' end. The colour map indicates the base-pairing probabilities, with ‘0’ being the lowest and ‘1’ being the highest. The 5’ and the 3’ ends are marked.

Figure S6:

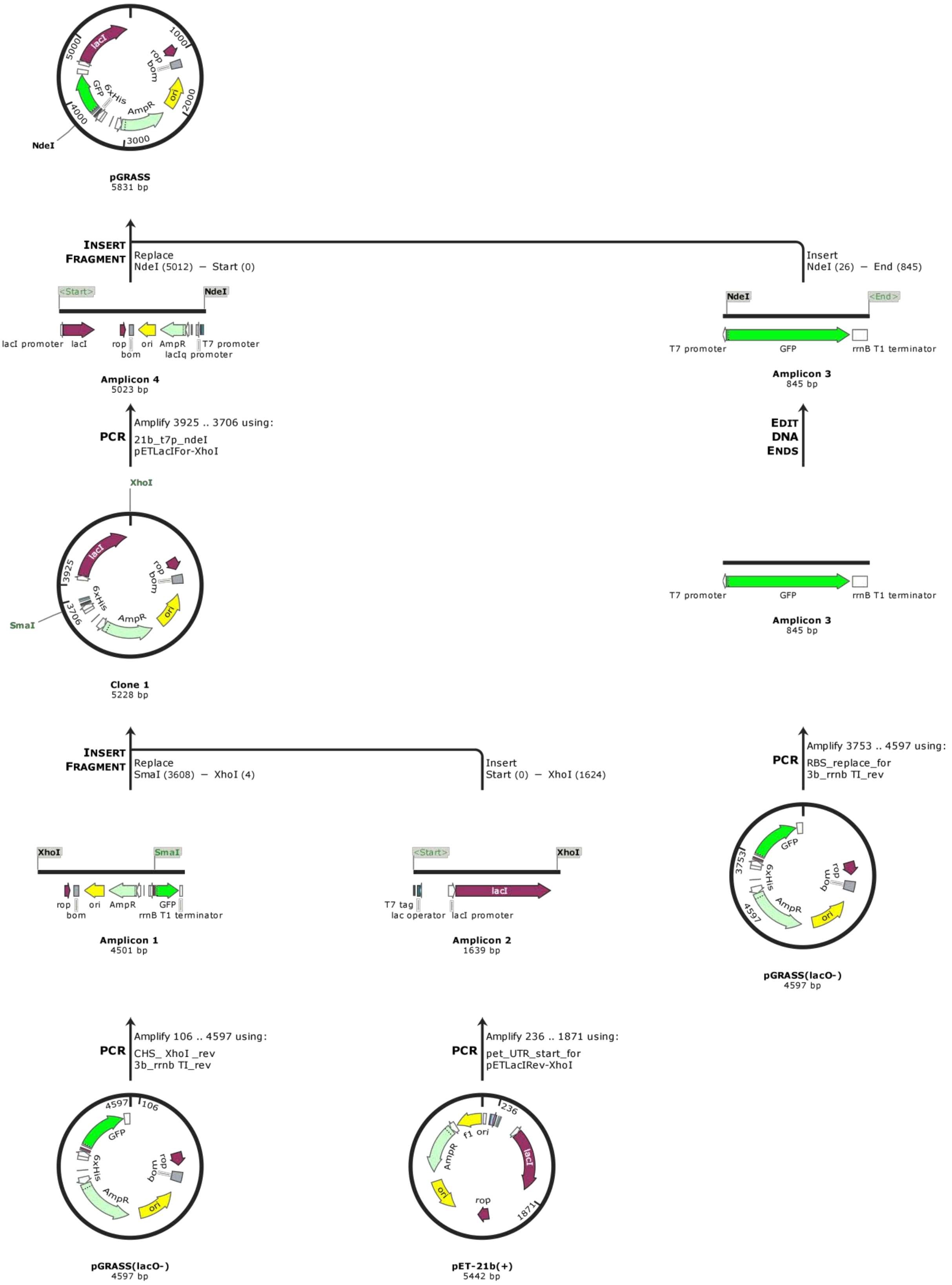

Figure S6: Schematic diagram of the steps involved in the generation of pGRASS from pGRASS(*lacO*-). Primers used in each step are annotated. The explanation of the schematic

starts from the bottom. To construct the vector, a XhoI site was first introduced pGRASS(*lacO*-) by PCR (Amplicon 1) with primers that incorporated the site. Next, the leader sequence of the T7 cassette as well as the *lacI* gene with its promoter was amplified from pET21b plasmid (Amplicon 2). Amplicon 1 was digested with XhoI and SmaI enzymes, whereas Amplicon 2 was digested with XhoI followed by ligation of the two. This construct was named clone 1. Since clone 1 was constructed by removing GFP from the parent plasmid, desired recombinants formed white colonies in a green background, and, hence, it was selected based on the absence of fluorescence. Thereafter, clone 1 was PCR-amplified with primers that incorporated an NdeI site after T7 promoter to produce Amplicon 4. *GFP* with its terminator was amplified from pGRASS(*lacO*-) thus yielding Amplicon 3, and was incorporated into Amplicon 4 through ligation at the NdeI and a blunt end. This final step generated pGRASS vector. Correct clones that formed green colonies in a white background were selected and sequenced to confirm the modifications.

Figure S7:

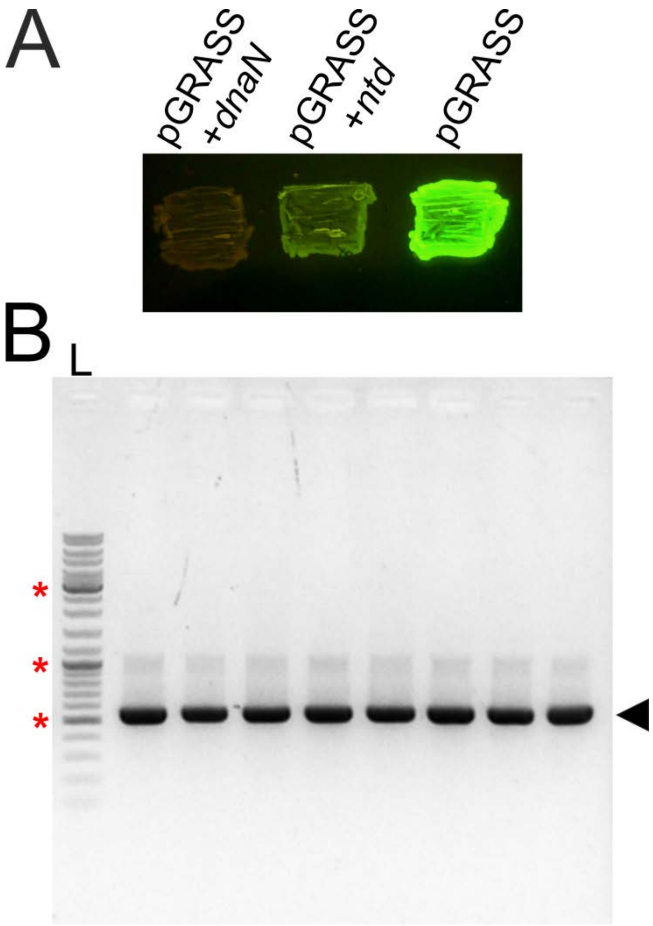

Figure S7: Cloning and selection of Lysin A N terminal domain (*lysA_ntd*) in pGRASS vector. Panel A shows the patch of the colony formed after ligation in the case of pGRASS+dnaN, pGRASS+ntd, and self-ligated pGRASS vector. *E coli* cells transformed with self-ligated pGRASS plasmid shows bright green fluorescence, whereas cells carrying pGRASS+*ntd* produce faint green fluorescence. Cells with pGRASS+*dnaN* are non-fluorescent. The image was captured after exposing the agar plate to blue light. Panel B shows the colony PCR from eight randomly selected non-fluorescent colonies from pGRASS+*ntd* transformation plate. Gene forward and vector reverse primers were used. The desired amplified band is marked with an arrowhead. Lane 'L' represents DNA ladder with few bands marked with '*', corresponding to 0.5, 1.0, and 3.0 kb size.
