# Supplementary material for: A positive Selection Escherichia Coli Recombinant Protein Expression Vector for One-Step Cloning
Source: Front Bioeng Biotechnol. 2022 Jan 3;9:776828. doi: 10.3389/fbioe.2021.776828 (PMC8761972; doi:10.3389/fbioe.2021.776828)
Supplement: Supplementary file 2 [file DataSheet1.PDF]

Supplementary tables

Supplementary Table S1: Analysis of the organisms from different domains of life for the presence of target codons in their genomes. Percentage of coding sequences (CDS), having at least one of the codons *viz*. TCA, TTA or CTA in their open reading frame (ORF) to enable selection in pGRASS, is given.

Organism	Percentage of CDS having at least one of TCA,TTA or CTA in the ORF
Escherichia phage T4	97.4
Tobacco mosaic virus	100
Human immunodeficiency virus 1	100
Mycoplasma genitalium	100
Escherichia coli	95.2
Bacillus subtilis	98.6
Mycobacterium tuberculosis	88.6
Dictyostelium discoideum AX4	99.6
Saccharomyces cerevisiae	99.3
Caenorhabditis elegans	97.1
Drosophila melanogaster	95.2
Arabidopsis thaliana	99.0
Danio rerio	98.8
Mus musculus	98.7
Homo sapiens	98.0

Supplementary Table S2: List of primers used in this study. The primers along with their nucleotide sequences are shown. The target reaction for each primer is also mentioned.

Primer	Sequence (5'-3')	Purpose
mtb_fr_DnaN	ATGGACGCGGCTACGACAAGAGTTGGC	Cloning of *dnaN* into pGRASS and pET21b
mtb_Re_DnaN	GCCCGGCAACCGAACCGGCATCAAC	
mdor_for	ATGCTGGACCCAGACGCGGCACGCGAGC	Cloning of *mdor* into pGRASS and pMS_QS_CHS
mdor_rev	ACCGCCTGAGGCCTGCATCAGCGTG	
adhE2_pMS_for	ATGCCTCAGACTGTGCGCGGTGTGATTTCTCG	Cloning of *adhE2* into pGRASS and PMS_QS_CHS
adhE2_pMS_rev	TCGGTCCAACACCACCACGGAGCGCAGC	
NTD_For	AGCATAGTCACACGCGACCACGCGCAG	Cloning *lysin_ntd* into pGRASS
NTD_rev	GCCGTCTTCAAGCTTCGCGTCCAGG	
pos_seq_for	CGGATCTTGGGGCTCATGTTAATACG	cPCR of clones in pGRASS(*lacO*-)
pos_seq_rev2	GCTCGTATAATGTGTGGAATTTCAC	
3b_rrnB-T1_rev	GTCGATTTGTCCTACTCAGGAGAGCGTTCACC	cPCR of clones in pGRASS
SDM_del1_CHS_LacIq	CAGGGTGGTGAATATGTTAATGGTGATGGTGATGGTGC	
ori_seq_rev	ACAGATCGCTGAGATAGGTGCCTC	Sequencing the *Ori* region in pGRASS(lacO-)
CHS_ XhoI _rev	CGGCTCGAGATCAGGGACAGCTTCAAGGATCGCTC	Amplification of Amplicon 1 for pGRASS construction
3b_rrnB-T1_rev	GTCGATTTGTCCTACTCAGGAGAGCGTTCACC	
pETLacIRev-XhoI	GTTGAAGGCTCTCGAGGGCATCGGTCGAG	Amplification of Amplicon 2 for pGRASS construction
pet_UTR_start_for	GGGCATATCTATATCTCCTTCTTAAAGTTAAACAAAATTATTTCTAGAGGG	

RBS_replace_for	AGGCCTATAGTGAGTCGTATTAACCATATG AGCAAGGGCGAGG	Amplification of Amplicon 3 for pGRASS construction
3b_rrnB-T1_rev	GTCGATTTGTCCTACTCAGGAGAGCGTTCA CC	
21b_t7p_ndeI	CCTTAAGCATATGCGCGAAATTAATACGAC TCACTATAGGGGAATTGTGAGC	Amplification of Amplicon 4 for pGRASS construction
pETLacIFor-XhoI	GCGGCGGTGCTCGAGGGCCTCAACCTACTA C	
ori_RT_for	GGCGCTTTCTCATAGCTCAC	qPCR primers targeting *ori* region in pGRASS and pET-3b
ori_RT_rev	AGTCGTGTCTTACCGGGTTG	
umuD_RT_frw	GTTGAACAGCGCATCGATCTGAATC	qPCR primers trageting of *umuD* gene in *E. coli* chromosome
umuD_RT_rev	GAATCACCACTTGCTTTGACGAAG	
